# Hyperoxia increases interleukin-17 in airway epithelial cells, alveolar type II cells and alveolar macrophages after ovalbumin-induced lung inflammation

**DOI:** 10.1186/1939-4551-8-S1-A50

**Published:** 2015-04-08

**Authors:** Fernando Monteiro Aarestrup, Akinori Nagato, Thais Martins, Keila Campos, Beatriz Aarestrup, Frank Bezerra

**Affiliations:** 1 Universidade Federal De Juiz De Fora, Brazil; 2 Federal University of Ouro Preto (UFOP), Brazil

## Background

There is no clear evidence on the pattern of inflammatory responses in exacerbation of neither asthma nor whether the hyperoxia is able to modify it. Because the hyperoxia induces reactive oxygen species and oxidative stress we believe this condition could interfere in the expression of interleukin-17 (IL17) in airway epithelial cells, alveolar type II cells and alveolar macrophages.

## Methods

The experimental design was approved by the Ethics Committee of Federal University of Ouro Preto (UFOP)-No.092/2012. Groups of female BALB/c mice (8 weeks old; 24.53±0.31g) were divided randomly in to five experimental groups as follows: Control group (CG) remained to air room; PBS group received aluminum hydroxide in phosphate buffered saline (PBS); OVA group (OVA) received ovalbumin and aluminum hydroxide in PBS; O2 group (O2) was exposed to 100% oxygen in a chamber for 24h; OVA+O2 group (OVA+O2) was exposed to 100% oxygen for 24h, received ovalbumin and aluminum hydroxide in PBS. The data were presented as the mean ± standard error of the mean. For continuous data, we used a One-Way Anova followed by the Student–Newman–Keuls post hoc test. For non-continuous data, we used the Kruskal–Wallis test followed by the Dunn’s post hoc test. In all instances, the significance level was set at 5% (p<0.05).

## Results

In bronchoalveolar lavage the hyperoxia decreases macrophage number in O2 (2.82±0.20) and OVA+O2 (1.72±0.15) and increases neutrophils number in O2 (1.79±0.13) and OVA+O2 (1.72±0.15), compared to CG (macrophage: 5.36±0.33) and (neutrophils: 0.02±0.00). The Lymphocytes number were higher in O2 (1.08±0.07) and OVA+O2 (0.97±0.08) compared to CG (0.43±0.02). When the animals were exposed to oxygen and ovalbumin, concomitantly (OVA+O2 - 4.55±0.23), the hyperoxia decreases lymphocytes number when compared to OVA (4.55±0.23). The TNF-alpha content were higher in PBS (134.00±7.03), OVA (126.30±3.40), O2 (141.60±6.08) and OVA+O2 (129.60±5.05) when compared to CG (94.67±2.03). In lung sections, the hyperoxia increases interleukin-17 in airway epithelial cells, alveolar type II cells and alveolar macrophages after ovalbumin-induced lung inflammation. When the animals were exposed to oxygen and ovalbumin, concomitantly, the staining with IL17 were increase when compared to OVA (p<0.001).

## Conclusions

Hyperoxia-induced oxidative stress increases IL17 in airway epithelial cells, alveolar type II cells and alveolar macrophages after ovalbumin-induced lung inflammation.

